# Timeliness and Completion Rate of Immunization among Nigerian Children Attending a Clinic-based Immunization Service

**DOI:** 10.3329/jhpn.v27i3.3381

**Published:** 2009-06

**Authors:** Ayebo E. Sadoh, Charles O. Eregie

**Affiliations:** Institute of Child Health, University of Benin, PMB 1154, Benin City, Edo state, Nigeria

**Keywords:** Immunization, Timeliness, Vaccination, Nigeria

## Abstract

To achieve maximal protection against vaccine-preventable diseases, a child should receive all immunizations within recommended intervals. Clinic records of 512 Nigerian children were evaluated for timeliness in receiving vaccines and the completion rates of the schedule. About 30% of the children presented after four weeks of age for their first immunization; 18.9-65% of the children were delayed in receiving various vaccines compared to the recommended ages for receiving the vaccines. Only 227 (44.3%) children were fully immunized. Health education and mass mobilization of the community and health workers are recommended to improve the uptake of vaccines and to encourage timely receipt of vaccines.

## INTRODUCTION

The standard measure of vaccination coverage is the percentage of children who have received the requisite number of vaccine doses irrespective of the age at receipt of the vaccine (1). However, to achieve maximal protection against vaccine-preventable diseases, a child should receive all immunizations within recommended intervals (2). Receipt of vaccines at recommended ages and intervals ensures that the child is adequately protected from target diseases at all times. The authors are aware of only one study that assessed the timeliness of receipt of vaccines in Nigerian children and only on a sample of 110 children (3).

Nigeria operates the immunization schedule of the Expanded Programme on Immunization which prescribes five visits to receive one dose of Bacille Calmette Guerin (BCG), four doses of oral polio vaccine, three doses of diphtheria, pertussis and tetanus vaccine, and one dose of measles vaccine (4). In 2004, the country included hepatitis B and yellow fever vaccines in its schedule, recommending the receipt of three doses of hepatitis B at birth, at six weeks of age, and at 14 weeks of age while yellow fever should be given at nine months of age, along with measles vaccine (5). Previous assessments of full immunization did not include hepatitis B and yellow fever (6,7).

This study evaluated the timeliness of receipt of immunization among children attending the immunization clinic of the Institute of Child Health, University of Benin, Benin city, Nigeria.

## MATERIALS AND METHODS

The Institute of Child Health immunization clinic offers services to inhabitants of Benin city, the capital of Edo state in mid-western Nigeria. The services offered include immunization, growth monitoring, nutrition education, and general health education. Immunizations are offered everyday, except for BCG and measles, which are offered only on Fridays. Every child who commences immunization in this facility has a record of his/her biodata and the date of receipt of various vaccines. About 1,000 children per year receive their immunizations in this facility. The immunization services offered were evaluated in terms of the timeliness of receipt of vaccines, the immunization rates for individual vaccines, including the recent additions to the schedule, and the completion rates of the infant immunization schedules

The study included 512 consecutive children who received their routine immunizations at the facility during September 2004–March 2005. Data on date of birth, age at commencement of immunization, place of birth, age of mother, age of father, and the date of receipt of various vaccines were retrieved from the clinic records of these children. Age at receipt of immunization was calculated in days using the dates of birth and the dates of receipt of vaccines. At the time of review of data (March 2007), the youngest child was aged at least 24 months. The number of visits made was also recorded. The uptake of vaccines was recorded as simple percentages.

The timeliness of receipt of a vaccine is determined by the recommended age for receipt of the given vaccine. Previous studies on timeliness, involving schedules recommending receipt of vaccines at specific dates, have allowed 14, 28, and 30 days of the grace period (2,3,7). In this study, four timeframes were used for evaluating the timeliness of receipt of three doses of oral polio vaccine (OPV1, OPV2, and OPV3), three doses of diphtheria, pertussis and tetanus vaccine (DPT1, DPT2, and DPT3), and three doses of hepatitis B vaccine (HepB birth, HepB2, and HepB3) as follows: (a) too early if the vaccine was received earlier than the recommended age; (b) being on time if the vaccine was received on or before two weeks after the due date; (c) acceptably early if received between two and four weeks after the due date, and (d) delayed if received after four weeks of the due date.

Full immunization was defined as receipt of BCG, three doses of OPV, three doses of DPT, three doses of hepatitis B vaccine, and a dose each of measles and yellow fever vaccines respectively.

Drop-out rate was calculated as the difference in percentage coverage in between the consecutive vaccines.

## RESULTS

There were 265 male and 246 female children. The sex of one child was not recorded. The mean age of the mothers was 28.35±5.14 (range 16-45) years while that of fathers was 35.4±7.05 (range of 19-80) years.

Table 1 shows the uptake of various vaccines. The uptake was the highest for BCG, OPV0, and HepB birth at 89.5%, 96.7%, and 93.8% respectively while it was the lowest for measles and yellow fever vaccines at 57.6% and 57.2% respectively. The uptake of HepB2 and HepB3 was 84.4% and 63.7% respectively. Compared to the uptake of DPT2 (81.1%) and DPT3 (69.5%) which should be received at the same ages, the difference was not significant (p<0.05). The full immunization status was achieved in 227 (44.3%) children.

**Table 1.** Uptake of vaccines and comparison of mean age at receipt of vaccines with recommended ages among 512 children attending a clinic-based immunization service

Vaccine	Uptake		Recommended age (days)	Age (days) at receipt			Difference∗ (days)	p value
	No.	%		Mean	Mode	Median		
BCG	452	88.3	0	27.9	10	18	27.9	0.0001
OPV0	493	96.3	0	27.5	10	17	27.5	0.0001
HepB B	480	93.8	0	44.0	43	43	44.0	0.0001
OPV1	449	87.7	42	66.1	43	43	24.1	0.0001
OPT1	460	89.8	42	59.3	43	47	17.3	0.0001
HepB2	432	84.4	42	83.6	71	75	41.6	0.0001
OPV2	400	78.1	70	100.3	71	86	30.3	0.0001
DPT2	416	81.1	70	92.3	71	80	22.3	0.0001
OPV3	305	59.6	98	133.9	99	117	35.9	0.0001
DPT3	356	69.5	98	126.8	99	112	28.8	0.0001
HepB3	326	63.7	98	143.9	134	124	45.9	0.0001
MEAS	295	57.6	270	287.4	271	280	17.4	0.0001
YF	293	57.4	270	287.8	271	280	17.8	0.0001

∗The difference between age at receipt of vaccines and recommended age for vaccine

HepB B=HepB birth; MEAS=Measles vaccine; YF=Yellow fever vaccine

Table 1 also shows the mean ages at receipt of different vaccines compared to the recommended ages. The difference between the mean ages at receipt and the recommended ages ranged from 17.4 days for measles to 45.9 days for HepB3. The deviation from the recommended ages was highly significant for all the vaccines (p=0.0001). There was increasing drop-out between consecutive vaccines, 7.2% between BCG and DPT 1, and 19.3% between DPT1 and DPT3. Between HepB birth and HepB3, the drop-out rate was 30.1% and 11.7% between DPT 3 and measles vaccine. The drop-out rate between OPV0 and measles vaccine was 38.7%. [OPV0 was used here rather than the standard BCG because it had the highest coverage of the three vaccines given at first contact].

Table 2 shows the age distribution at receipt of the vaccines recommended at birth. Less than 50% of the children received the doses of vaccines to be received at birth within the first two weeks of life. Table 3 shows the timeliness of receipt of the various vaccines; 18.7-61.5% of the children received various vaccines on time while 18.9-65% were delayed in receipt of the various vaccines. Children were more likely to receive DPT1 on time while more children were delayed for receipt of hepatitis B vaccines, with 65% being delayed for receipt of HepB2 and 63.8% for HepB3.

**Table 2.** Age distribution of 512 children at receipt of BCG, OPV0, and HepB birth

Age (days) at receipt	BCG		OPV0		HepB birth	
	No.	%	No.	%	No.	%
≤14	208	42.2	170	37.6	92	19.2
15-28	131	26.6	142	31.4	68	14.2
≥29	154	31.2	140	31.0	320	66.6

**Table 3.** Timeliness of receipt of vaccines among 512 children attending a clinic-based immunization service

Vaccine	Timeliness of receipt of immunization							
	Too early		On time		Acceptably early		Delayed	
	No.	%	No.	%	No.	%	No.	%
OPV1	30	6.7	232	51.7	49	10.9	138	30.7
DPT1	37	8.1	283	61.5	53	11.6	87	18.9
HepB2	12	2.8	94	21.8	45	10.4	281	65.0
OPV2	16	3.8	175	43.8	69	16.0	146	36.4
DPT2	24	5.8	215	51.8	69	16.6	107	25.8
OPV3	12	3.9	118	38.7	68	19.0	117	38.4
DPT3	16	4.5	166	46.7	71	19.9	103	28.9
HepB3	18	5.5	61	18.7	39	12.0	208	63.8

The large majority (n=213; 73.2%) of the children received their measles vaccine at nine months of age while 33 (11.3%), 14 (4.8%), and 6 (2.1%) received it at 10, 11, and 12 months or later respectively. Some (n=20; 6.8%) children received measles vaccine before the age of nine months while the date of receipt was not recorded for five children who had received measles vaccine, and they were, therefore, excluded from this analysis.

The age at commencement of immunization was significantly associated with the immunization status. Of those who commenced immunization after the age of 28 days, 99 (66.9%) did not complete their immunization compared to 186 (51.1%) of those commencing before 28 days. Commencement of immunization at an age above 28 days was, thus, associated with a higher chance of non-completion of the immunization schedule (p=0.01). Age of mother and father and place of birth were not significant as determinants of completion of the immunization schedule.

## DISCUSSION

The uptake of vaccines for the study population was higher than for the national figures (5). The uptake of earlier vaccines in the schedule was higher than for latter vaccines. This was consistent with findings of other studies (8-10). However, the fact that none of the vaccines received at first contact was 100% may indicate the presence of missed opportunities. It was also observed that the uptake of the three vaccines at first contact was different. This was probably due to non-administration of simultaneous vaccines with the rescheduling of BCG which is given only on Fridays.

The uptake of hepatitis B vaccine was not significantly different from that of DPT which suggests that, as a new vaccine, its performance was adequate. However, the target as defined in the first EPI regional strategic plan for 2001-2005 for the African region of HepB3 coverage being equal to that of DPT3 was not met (11). The target of 80% coverage for DPT3 was also not met (11). High drop-out between DPT1 and DPT3 and between HepB birth and HepB3 may be responsible for this. It was observed that there was a tendency of delay in receipt of hepatitis B vaccine doses. Late presentation for immunization among the study population necessitated the synchronization of the second dose of hepatitis B vaccine with the second dose of DPT, resulting in separate appointments for DPT3 and HepB3. These separate appointments may have resulted in increased drop-out for HepB3 which was scheduled later. Non-administration of simultaneous injections was also a contributory factor to delay in receipt of hepatitis B vaccine doses and may also have contributed to higher drop-out.

There was a steady increase in drop-out between consecutive vaccines, with drop-out being the highest between DPT3 and measles vaccine. This finding has been previously documented (7,12). The suggested explanation included the longer interval between DPT3 and measles vaccine (three and a half months) compared to that between the earlier vaccines in the schedule (four weeks). It is also suggested that, as the number of weeks/months postpartum increase, mothers begin to be engaged in other activities such that they may forget and/or may not have time to make scheduled visits for immunizations.

The full immunization rate for the children attending the study facility was only 44.3%. This is low compared to the findings of another clinic-based study from Benin, in which 72.4% of infants studied had completed the immunization schedule (7). The difference may stem from the fact that immunization is free for this study population whereas mothers had to pay for consumables in the other clinic which is also privately-owned. These other mothers may, thus, be more motivated. The other study evaluated children during their visit for the last vaccine in the series. This would have excluded children who defaulted completely at earlier ages, giving an erroneous picture of a higher completion rate for the schedule. Also, the inclusion of hepatitis B and yellow fever vaccines in the definition of full immunization may have contributed to the lower rate in this study as this is a more stringent criterion.

From this study, it is observed that children presented late for immunization, with over 30% presenting after four weeks of life. This late presentation contributed to delay in receipt of the earlier vaccines in the schedule. The age at presentation was also a significant determinant of completion of the schedule. Children who presented early were more likely to complete the immunization schedule compared to those who presented late. This finding has previously been reported (13). The reasons for the delay in commencing immunization were not explored in this study.

For the vaccines administered at birth, less than 50% of the children presented within the first two weeks of life. The birth doses of vaccines are to prevent perinatal or early neonatal transmission; thus, the late presentation of children for these doses would place them at a risk of contracting the target diseases before receipt of the requisite immunization. This is particularly critical for hepatitis B immunization for which its efficacy in preventing perinatal transmission is dependent on the administration of the vaccine within 24 hours of birth (14).

The immunization schedule defines minimal age for receipt of vaccines. In this study, 57-75% of the children received various vaccines on time or acceptably early, except for HepB2 and HepB3 for which the corresponding values were 32.4% and 30.7% respectively. This means that a significant proportion of the children did not receive their vaccines on time or early enough, thus leaving them unprotected or inadequately protected for varying periods of time. In the Nigerian study that examined age-appropriate vaccinations, only 26% of surveyed children received all their vaccines within the recommended period (3). Also in the United States, approximately 74% of children were delayed for one or more vaccinations during the first 24 months of life (1).

The implication of delay in receipt of vaccines is that a pool of children with incomplete or no immunization may build up (albeit temporarily). The presence of such a pool of susceptibles may predispose to the spread of an epidemic. While the specific reasons for the delay in receipt of vaccines were not explored, it is possible that the delay in commencing immunizations, as earlier explained, was a contributing factor. This explanation seems tenable against the backdrop that most delays were for earlier vaccines in the schedule.

Almost three-fourths of those who received measles vaccine did so in the ninth month of life. The timely receipt of this vaccine may be because it is the last vaccine in the immunization schedule, and mothers are more likely to remember this. It may also be a reflection of the commitment of this group of mothers who presented for the last vaccine of the schedule, especially when considered against high drop-out between OPV0, one of the first vaccines in the schedule, and the measles vaccine, the last one in the schedule. The uptake for this vaccine is, however, low, with just over 50% of the study population receiving it.

The study had a few limitations. There was no tracking of the children to determine if they had moved out of the city or if they had completed their immunizations with a different provider. The data were also not checked against the immunization cards of the children to verify the completeness and accuracy of the records.

Based on the findings of the study, the following recommendations are made:

Health education and mass mobilization of the community served by this clinic are urgently needed not only to increase the uptake but also for the timely receipt of vaccines. Early commencement of immunization and completion of the schedule should be emphasized. Health workers would need to be trained to implement the practice of administering birth doses of hepatitis B vaccine, OPV, and BCG while strategies to reach babies born outside health facilities with doses of vaccines at birth should be explored. This is especially so given the current impetus to eradicate poliomyelitis and the fact that hepatitis B vaccine needs to be given within 24 hours of birth for it to be effective in preventing perinatal transimission. This clinic should incorporate regular evaluations of its practice to enable it to improve its effectiveness. Finally, a community-based evaluation of immunization is required to further elucidate the factors affecting the uptake of vaccines, including the age at presentation for vaccinations.

## ACKNOWLEDGEMENTS

The authors thank the Institute of Child Health academic staff for their contributions in designing the immunization register and for ensuring data collection. The authors also thank Mrs. G. Akpughe and Ms Enite Odeka for assisting with data entry into the immunization registers. The authors also thank the nurses Mrs. B. Onoguwe, Mrs. D. Ogbeide, and Mrs. T. Eyakwanor for their care of the study children and for their supervision of data entry.
